# Incidental Intrathecal Injection of Meglumine Diatrizoate

**DOI:** 10.5812/ircmj.9661

**Published:** 2014-05-05

**Authors:** Mansour Masjedi, Abbas Khosravi, Golnar Sabetian, Mohammad Reza Rahmanian

**Affiliations:** 1 Shiraz anesthesiology and critical care research center , Shiraz University of Medical Sciences, Shiraz, Iran; 2 Intensive care department , Iranian Hospital – Dubai , Dubai , UAE; 3 Anesthesia department , Shiraz University of Medical Sciences, Shiraz, Iran

**Keywords:** Contrast Media, Edema, Brain, Intracranial Pressure

## Abstract

**Introduction:**

Myelograghy is a process of instilling contrast medium to the subarachnoid space for evaluating the spinal column by radiography. There are various contrast solutions for different radiographic studies but not all of them are suitable for spinal column evaluation.

**Case Presentation:**

Our patient was a 60-year-old man who developed severe pain, tonic clonic convulsions and cardiopulmonary arrest after intrathecal injection of 14 mL of meglumine diatrizoate during an elective myelography procedure. Many of these cases would die or suffer from permanent sequelae if appropriate treatment is not received.

**Conclusions:**

Our subject recovered completely without any sequelae after receiving appropriate treatment in a multidisciplinary intensive care unit.

## 1. Introduction

The human subarachnoid space is a poor depository for foreign substances and approaching to this space can accompanied by numerous complications. Despite this, Myelography has been an important diagnostic tool for evaluating the spinal column pathway since the 20th century. Even the safest myelographic agent can ignite a disaster, besides, wrong agent, wrong concentration, and wrong approach can increase the chance for developing severe complications, and permanent sequelae. Fortunately, nowadays the number of invasive diagnostic myelography procedures has been reduced dramatically which is due to advances in noninvasive body imaging like MRI (Magnetic Resonance Imaging).

## 2. Case Presentation

**Figure 1.**
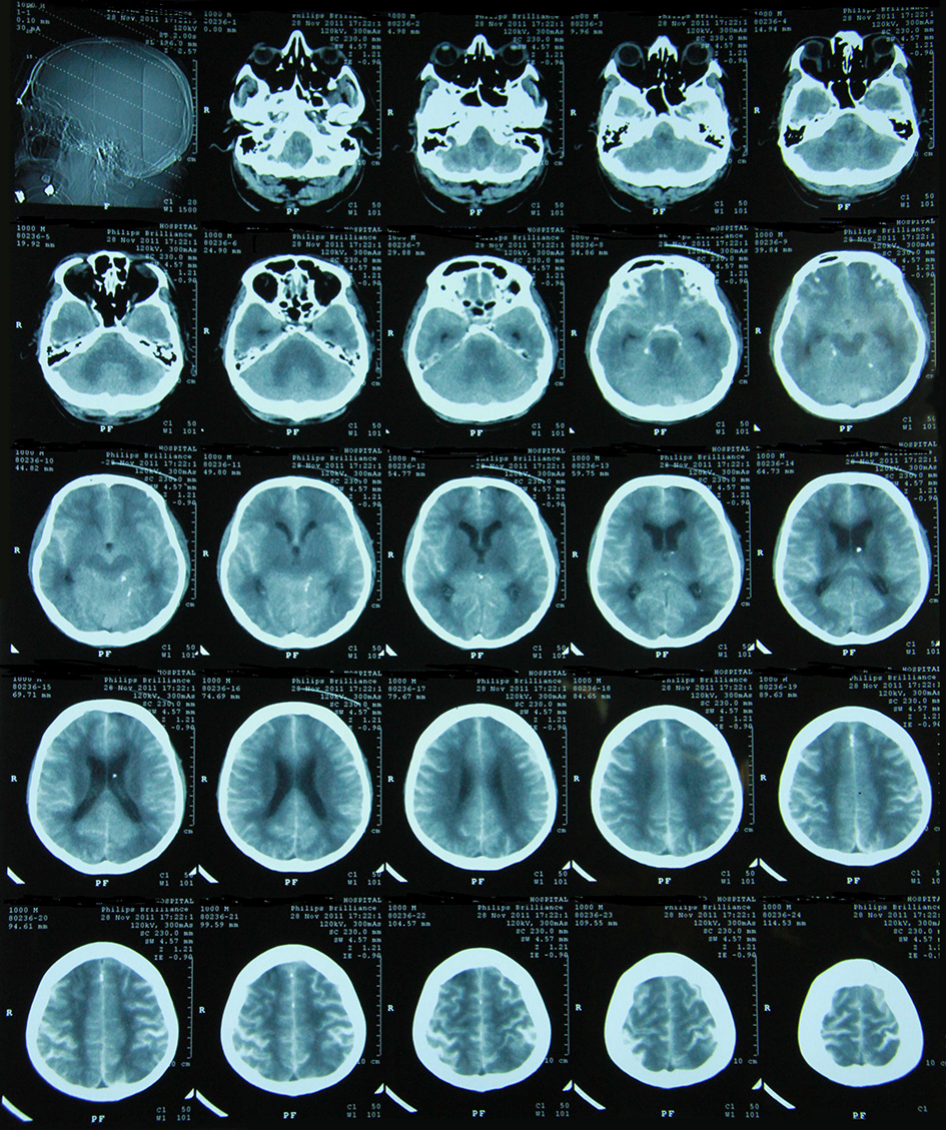
Computerized Brain Scan, Showing Severe Brain Oedema, Hyper-Dense Area After Injection of Meglumine Diaterizoate

A 60-year-old man, who worked as Hospital General Director, known case of war injured patient with multiple metallic missile fragments in different parts of his body, developed clinical symptoms of spinal canal stenosis (numbness and weakness of lower extremities) for which referred to radiology department for myelography by Neurosurgeon since diagnostic MR was not possible due to the embedded metallic fragments in the body. He had positive medical history for multiple surgical procedures and 5 days ICU admission after laparotomy due to severe undiagnosed illeus after herniorrhaphy. There was no known history of sensitivity to any food or medication in the past. Intrathecal injection performed by anesthesiologist using a 20 Guage disposable spinal needle. Intrathecal injection done at the level of L4–L5 and 14 mL of Meglumine 75% injected into the subarachnoid space. Patient was stable for 40 minutes when he started complaining of intolerable pain and muscle spasm in lower extremities which forced anesthesiologist to intubate and sedated the patient in order to control pain and muscle spasm and patient was transferred to intensive care unit with impression of hypersensitivity to contrast medium injection. First brain CT scan (3 hours post intrathecal injection) showed signs of high ICP, dilated ventricles, severe hydrocephaly and presence of contrast medium in all arachnoidal spaces. Ischaemic change could not be ruled out by radiologist. Ventriculostomy catheter was inserted in right lateral ventricle and patient kept under direct supervision of Intensive care consultants with their multidisciplinary strategy. As reported by neurosurgeon, brain was very edematous and the first measurement of ICP shows 40 cmH2O. In ICU, patient was kept fully sedated with infusion of Midazolam (5 mg/h) and propofol (100-200mg/h). Pupils were 2-3 mm in size with sluggish response to the light. Mean ICP was kept between 10–15 cm water and Cerebral perfusion pressure (CPP) maintained above 70 mmHg. Patient was put on mechanical ventilator- SIMV mode and Arterial Blood Gas parameters were acceptable during his ICU stay. Patient was hemody namically stable with MAP (Mean Arterial Pressure) above 65 mmHg. Follow up CT Brain scans showed gradual reduction of brain edema and disappearance of contrast medium from arachnoidal spaces (Figure 1). Sedative drugs were gradually tapered after 3 days and patient regained his consciousness and extubated successfully after 7 days. He got discharged from hospital after 10 days without any sequelae.

## 3. Discussion

Nowadays, lumbar myelography is not very common due to (serious complications like neurotoxicity (1), encephalopathy (2), cerebral edema (3), selective cortical injury (4), and transient partial amnesia (5) Magnetic Resonance Imaging. Although, an intact blood–brain barrier appears to protect the nervous system, some agents even though safe to give intravenously, being neurotoxic should not be instilled intrathecally (6). Donaghy et al. suggested that disruption of the blood-brain barrier occurs during angiographic use of nonionic contrast, allowing diffusion into the parenchyma, thus exerting a neuronal toxic effect resulting in acute encephalopathy (7). Oil-based agents (e.g. iophendylate) were introduced many years ago but these agents were suboptimal (8) and researchers found some of these materials like Iopromide can produce the highest degree of neurotoxicity (9). Some authors consider the neurotoxicity as secondary to the volume of contrast medium used, as well as to the patient’s history of hypertension, which predisposed him to disruption of the BBB and cerebral autoregulatory dysfunction (10). Several case reports have documented neurotoxicity following angiography using nonionic contrast media, including encephalopathy (2, 3), cerebral edema (3), selective cortical injury (4), and transient partial amnesia (5). Later, ionic water-soluble media were developed. However, they are unsuitable for direct contact with neural tissue and the neurotoxicity and other complications of these agents like: severe muscle spasms, seizures, cerebral edema and hemorrhage, coma, paralysis, hypotension, hyperthermia, rhabdomyolysis, multisystem organ failure, and even death are well documented (11-14). Clonic convulsions of the lower extremities after intrathecal injection of meglumine iothalamate and meglumine iocarmate, have been reported by different authors and the possibility of late sequelae, due to adhesive arachnoiditis must be seriously considered (9). Animal experimental and preliminary clinical reports show that metrizamide (Amipaque) which was introduced in 1969 as the first nonionic water soluble contrast, is less toxic in the subarachnoid space than commercially available water-soluble contrast media (9, 15). Current theories of the pathophysiology of neurotoxicity due to intrathecal use of nonionic contrasts include direct neurotoxicity (16), serum osmolarity differences, (10) and lipid solubility of the agent (17). We believe that the mechanism of acute onset of massive cerebral edema in our patient was the injection of high dose of Meglumine (14 mL) and dysfunction of the patient’s BBB, with subsequent loss of autoregulation of cerebrovascular blood flow which leads to vasogenic brain edema; and we emphasise that immediate recognition of a mistake and prompt aggressive treatment was life saving. However, general measures include avoiding dehydration and systemic hypotension, maintaining normal body temperature, and positioning the head and neck so that intracranial venous outflow obstruction is prevented. Actually injection of meglumine diatrizoate, which is an ionic contrast agent, into the subarachnoid space must be avoided, since even small amounts may produce convulsions and possible fatal reactions. Specific therapeutic interventions like ICP monitoring, osmotherapy, controlled hyperventilation, administering corticosteroids or diuretics, and suppressing cerebral metabolism should be considered too (18). In severe cases, craniotomy and insertion of ventricular drain are the only choice. Adverse reactions to contrast agents can be mild to life threatening; so, focusing on the changes in the level of consciousness and evaluating any new or worsening of neurological symptoms are important. Admission in Intensive care unit, close monitoring, Serial CTs and MRIs are helpful to manage these patients and thereby saving lives.
